# Utility of Brainstem Auditory Evoked Response as a Diagnostic Tool and Rituximab as a Treatment for Severe Bickerstaff Brainstem Encephalitis: A Case Report

**DOI:** 10.7759/cureus.57993

**Published:** 2024-04-10

**Authors:** Myra T Aninang, Marianne Rae Baltazar-Libiran, Ludwig F Damian

**Affiliations:** 1 Neurology, Institute for Neurosciences, St. Luke's Medical Center, Quezon City, PHL

**Keywords:** guillain-barré syndrome (gbs), neuroimmunology, anti-gq1b antibody syndrome, brainstem auditory evoked potential, rituximab therapy, bickerstaff brainstem encephalitis

## Abstract

Bickerstaff brainstem encephalitis (BBE) is a rare disorder that is characterized by ophthalmoplegia, ataxia, and disturbance in consciousness. Definite diagnosis is made primarily through clinical presentation and serology testing with anti-GQ1b antibody. However, in a country where access to serologic testing is scarce, electrophysiologic tests such as brainstem auditory evoked response (BAER) may contribute to the diagnosis. Due to its rarity and generally good prognosis, there is no established consensus for the treatment of BBE. Immunomodulatory treatments such as intravenous immunoglobulin (IVIG), plasma exchange, steroids, or a combination of these therapies are often used with good response. However, there are severe cases that respond poorly to these conventional treatments.

We report the case of a 26-year-old Filipino man who came in for sudden onset of diplopia, with a one-week history of upper respiratory tract infection. Subsequently, he developed paresthesias, quadriparesis, and an altered level of consciousness. On initial examination, he only had partial third nerve palsy, but eventually became quadriparetic and obtunded during admission. Initial electromyography and nerve conduction velocity (EMG-NCV) study showed a reduced recruitment pattern of the right rectus femoris, absent H reflexes of bilateral posterior tibial nerves, and no abnormal increase in temporal dispersion. Cranial MRI with contrast was unremarkable. Video electroencephalogram (video-EEG) showed intermittent generalized 5-6 Hz and 6-7 Hz theta slowing of the background activity in the stimulated state. BAER was done revealing bilateral partial dysfunction of the auditory pathways to support brainstem involvement of the disease. He received IVIG and methylprednisolone pulse therapy with no significant clinical improvement. Hence, he was given a rituximab infusion. One week post-rituximab, he had sustained wakefulness and was able to move his extremities.

## Introduction

Bickerstaff brainstem encephalitis (BBE) is a neuro-immunologic disorder involving both the peripheral and central nervous system. It is defined by its acute-onset, progressive, relatively symmetric external ophthalmoplegia, ataxia, and altered level of consciousness [1]. Some patients with intact consciousness may present with extensor plantar response and hemisensory loss, suggestive of central involvement [2]. It forms a spectrum of post-infectious demyelinating disorders with other diseases such as Guillain-Barrè syndrome (GBS) and Miller-Fisher syndrome (MFS).

In 2012, the diagnostic criteria for BBE was established. It divided the diagnosis into definite and probable based on the presence of typical neurological triad (external ophthalmoplegia, ataxia, and impaired level of consciousness) and serum IgG anti-GQ1b antibody status [3]. Other tests such as cranial MRI, cerebrospinal fluid (CSF) studies, and electroencephalogram (EEG) were primarily used to help rule out other conditions. Electrophysiologic tests such as brainstem auditory evoked response (BAER), somatosensory evoked potential (SSEP), and electromyography and nerve conduction velocity (EMG-NCV) may aid in establishing a diagnosis but are not included in the current diagnostic criteria.

There is no standard of treatment for BBE yet, but due to its hypothesized immune-related pathophysiology, immunotherapies such as intravenous immunoglobulin (IVIG), steroids, and plasma exchange are often given with favorable outcomes. However, there were reported cases when the clinical response from these therapies was inadequate. Rituximab is a monoclonal antibody that targets CD20 proteins in our immune cells. It has been utilized in various lymphoproliferative disorders and autoimmune conditions and has been reported to be effective in severe cases of BBE.

## Case presentation

We present the case of a 26-year-old male who came in due to diplopia. He had a history of upper respiratory tract infection one week before his neurologic symptoms. At the emergency department, physical examination only showed limitation of elevation of the left eye. A plain cranial MRI was requested which revealed no acute lesions. The respiratory panel tested positive for influenza A infection. He was subsequently admitted as a case of possible brainstem encephalitis. Later that day, he complained of generalized paresthesia, weakness, and difficulty swallowing. Upon re-examination, he had incomplete bilateral third and sixth nerve palsy, facial diplegia, mild dysarthria, flaccid quadriparesis, truncal ataxia, and areflexia.

On the second hospital day, the EMG-NCV study showed a reduced recruitment pattern of the right rectus femoris and absent H reflexes of bilateral posterior tibial nerves, without an abnormal increase in temporal dispersion (Table 1 and Table 2). Post-procedure, he became obtunded and was intubated for airway protection. Assessment during this time was possible BBE. Lumbar puncture was facilitated which showed albuminocytologic dissociation; cerebrospinal fluid (CSF) cell count was normal, with elevated CSF protein (Table 3). Infectious work-up including CSF meningitis-encephalitis panel, bacterial, fungal, and *M. tuberculosis* cultures, and autoimmune tests such as antinuclear antibodies (ANA), erythrocyte sedimentation rate (ESR), C-reactive protein (CRP), antineutrophilic cytoplasmic antibody (ANCA), and anticardiolipin antibodies were all unremarkable. IVIG infusion at a dose of 2 grams per kilogram for five days was started. Interim, there was further deterioration in consciousness; his pupils were 3 mm, with intact pupillary light reflex, but absent bilateral oculocephalic and corneal reflexes. A 2-hour video-EEG was done to help localize and investigate the etiology of the altered consciousness. It showed intermittent diffuse 5-6 and 6-7 Hz theta activity on stimulation and maintenance of sleep patterns.

**Table 1:** Nerve conduction velocity studies done on day 2 vs day 7 of illness SAP: sensory nerve action potential measured in µV, microvolts; MCV: motor conduction velocity measured in m/s, meters/seconds; distal latency measured in ms, milliseconds; CMAP: compound muscle action potential measured in mV, millivolts

Nerve study	D2 of illness	D7 of illness	D2 of illness	D7 of illness
	Left extremity		Right extremity	
Median nerve				
SAP (index-wrist)	17 µV, 56 m/s	6 µV, 58 m/s	17 µV, 53m/s	Not done
SAP (palm-wrist)	31 µV, 55m/s	Not done	53 µV, 51 m/s	
SAP (wrist-elbow)	18 µV, 64 m/s		13 µV, 68 m/s	
MCV	56 m/s	63 m/s	64 m/s	
Distal latency	3.2 ms	3.7 ms	3.2 ms	
CMAP (wrist)	15.1 mV	3 mV	12/5 mV	
CMAP (elbow)	14.7 mV	3.3 mV	11.9 mV	
F-wave to abductor pollicis brevis	23.8 ms	No clear F-wave	23.9 ms	
Ulnar nerve				
SAP (digit 5-wrist)	8 µV, 56 m/s	No response	12 µV, 51 m/s	Not done
MCV (forearm)	57 m/s	68 m/s	67 m/s	
MCV (across elbow)	55 m/s	62 m/s	62 m/s	
Distal latency	3.0 ms	2.9 ms	3.6 ms	
CMAP (wrist)	11.2 mV	3.6 mV	10.5 mV	
CMAP (elbow)	10.8 mV	3.5 mV	10.4 mV	
CMAP (above elbow)	7.1 mV	1.8 mV	8.8 mV	
F-wave to abductor digiti minimi	24.8 ms	No clear F-wave	23.4 ms	
Radial nerve				
SAP	21 µV, 55 m/s	No response	22 µV, 51 m/s	Not done
Sural nerve				
SAP	21 µV, 54 m/s	No response	27 µV, 53 m/s	No response
Common peroneal nerve				
SAP	12 µV, 47 m/s	Not done	12 µV, 47 m/s	Not done
MCV (leg)	56 m/s	41 m/s	55 m/s	40 m/s
MCV (across knee)	52 m/s	41 m/s	53 m/s	42 m/s
Distal latency	4.2 ms	5.9 ms	3 ms	3.1 ms
CMAP (ankle)	4.7 mV	0.1 mV	3.5 mV	1.1 mV
CMAP (knee)	4.6 mV	0.1 mV	3.4 mV	0.1 mV
CMAP (above knee)	4.3 mV	0.7 m	2.8 mV	0.4 mV
Post. tibial nerve				
MCV	50 m/s	53 m/s	56 m/s	51 m/s
Distal latency	3 ms	4 ms	3.9 ms	4.3 ms
CMAP (ankle)	15.5 mV	7.6 mV	14 mV	5.2 mV
CMAP (knee)	13.6 mV	5.7 mV	12.3 mV	4.3 mV
F-wave to abductor hallucis	43.2 ms	No clear F-wave	43.6 ms	No clear F-wave
H-reflex	No response	No response	No response	No response

**Table 2:** Electromyography done on day 2 of illness PSW: positive sharp waves

Muscles	Insertional activity	Spontaneous activity			Motor unit potentials
		Fibrillation	Fasciculation	PSW	
Right biceps	Normal	Absent	Absent	Absent	Normal motor unit potential, amplitude, morphology, and duration. Normal recruitment pattern
Right first dorsal interosseous	Normal	Absent	Absent	Absent	Normal motor unit potential, amplitude, morphology, and duration. Normal recruitment pattern
Right gastrocnemius (medial)	Normal	Absent	Absent	Absent	Normal motor unit potential, amplitude, morphology, and duration. Normal recruitment pattern
Right rectus femoris	Normal	Absent	Absent	Absent	Reduced recruitment pattern

**Table 3:** Lumbar puncture done on day 3 of illness CSF: cerebrospinal fluid; IgG: immunoglobulin G *Values based on St. Luke's Medical Center Global City Institute of Pathology laboratory reference range

CSF studies	Values	Reference range
Opening pressure	18 cmH20	10-18 cmH20
CSF/serum glucose ratio	0.62	>0.6
CSF protein	74 mg/dl	8-32 mg/dL*
Total cell count	4 cells/µL	0-5 cells/µL
White blood cells	4 cells/µL (lymphocytes 100%)	0-5 cells/µL
Red blood cells	0 cells/µL	0-5 cells/µL
Meningitis and encephalitis panel bacterial, MTB, and fungal cultures	Negative	-
Cell block and cytology	Acellular	-
CSF IgG	2.09	0.48-5.86 mg/dL*
CSF oligoclonal panel	Present	-

**Figure 1:**
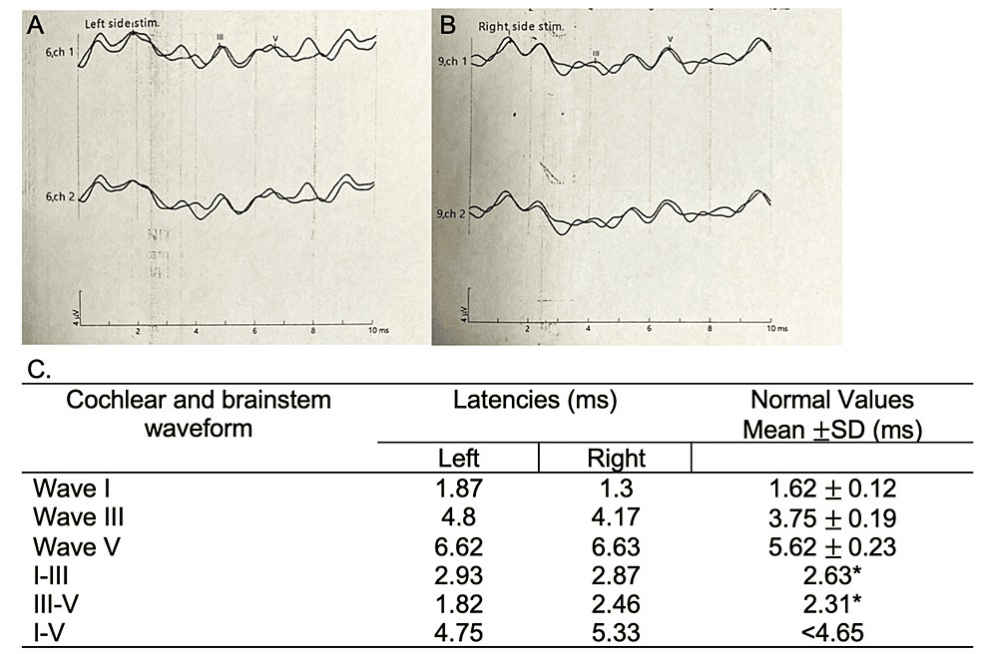
Brainstem auditory evoked response done on day 10 of illness A: waveform from the left ear; B: waveform from the right ear; C: cochlear and brainstem waveform latencies in milliseconds (ms) *Upper limit normal Normal values based on St. Luke's Medical Center Global City Institute of Neurosciences laboratory reference range Stimulation of both ears individually using click stimulus polarity at 90 dB evoked well-defined cochlear and brainstem waveforms I, III, and V, both ipsilaterally and contralaterally with bilateral ear stimulation

On the sixth hospital day, he completed five days of 140 grams IVIG without significant clinical response. He was then started on methylprednisolone pulse therapy at 500 milligrams IV every 12 hours for three days. Repeat cranial MRI did not show any significant enhancement or T2 hyperintensities, especially in the brainstem. On day 7, a repeat NCV study (Table 1) showed decreased median nerve sensory amplitudes and absent response from the left radial, ulnar, and bilateral sural nerves. There were also low motor amplitudes in both median, ulnar, peroneal, and tibial nerves with absent F-wave responses. These findings showed an acute progressive sensorimotor symmetric polyneuropathy which was suggestive of acute motor and sensory axonal neuropathy (AMSAN) variant of acute inflammatory demyelinating polyneuropathy (AIDP). BAER showed bilateral partial dysfunction of the auditory pathways supporting central conduction dysfunction at the level of the pons (Figure 1). 

There was no significant clinical improvement; hence, on the 11th hospital day, the patient was given rituximab 500 milligrams IV infusion. On day 1 post-treatment, he showed brief movements of his lower extremities. Repeat EEG showed improved background activity, with more frequent 6-7 Hz theta activity and occasional generalized low voltage 18-22 Hz beta activity. On day 3 post-rituximab, he had partial eye opening, and on day 6, he was able to sustain wakefulness.

The patient was weaned off the mechanical ventilator and transferred out of the ICU on day 20 of admission. BAER was repeated which showed normal results with improved latencies. He was eventually discharged on day 47 of admission, with a decannulated tracheostomy, able to feed orally, and ambulate with support.

## Discussion

We have described the case of a Filipino patient presenting with the triad of symptoms of BBE, diagnosed with the aid of BAER, and who showed significant improvement with rituximab infusion, six days after completion of IVIG and two days post-methylprednisolone therapy.

In the early 1950s, Bickerstaff and Cloake reported three cases presenting with ophthalmoplegia, ataxia, and drowsiness which is later termed BBE. The worldwide incidence of this disease has not been established, but it is found to be most common in Japan where the annual occurrence is approximately 100 cases per year [4]. In the Philippines, there are only three published case reports of BBE. The first two patients, reported by Vatanagul et al. and Ando and Belonguel, both presented with ophthalmoplegia and ataxia, followed shortly by altered mental status [5,6]. The third patient reported by Cabungcal and Pabellano-Tiongson presented with ascending bilateral weakness of extremities followed by a decline in consciousness, ptosis, and ophthalmoplegia. Post-mortem autopsy revealed demyelination on the pons and sural nerve which further supports its combined central and peripheral demyelinating pathology [7]. Only the first patient underwent serologic testing and tested positive for serum anti-ganglioside antibodies specifically GM1b IgG and GQ1DG. Similarly, our patient presented with the triad of symptoms and, like the other two reported cases, did not have serologic testing for anti-GQ1b antibody.

The disease pathophysiology remains poorly understood and is hypothesized to be triggered by an infectious process because of the frequent antecedent history of infection. As discoveries on its immunological mechanism are being uncovered, its diagnostic criteria evolved. The most recent classification highlights the importance of IgG type Gq1b antibody in establishing its definite diagnosis (Table 4) [3]. However, it does not include electrophysiologic studies such as EMG-NCV, BAER, or SSEP. In the Philippines, serologic testing for anti-ganglioside antibodies such as anti-GQ1b is not available locally. For our patient, other tests such as EEG and BAER were done to investigate for CNS lesions that may be radiographically occult. BAER is a test of auditory brainstem function to screen for lesions from the auditory nerve up to the inferior colliculi of the midbrain. This noninvasive test has been utilized in diagnosing sensorineural hearing loss and auditory neuropathy and allows the localization of brainstem lesions, by pinpointing the location and nature of the impairment along the auditory pathway [8]. Our patient's result showed central conduction dysfunction at the level of the pons, therefore helping establish the involvement of the brainstem. A similar case in Japan showed the utility of BAER in establishing the diagnosis of BBE in the acute phase in an elderly woman who presented with dizziness, weakness, and impaired consciousness with a preceding history of upper respiratory tract infection. Like our patient, there were no significant findings in the cranial MRI, EEG, or CSF examinations. Her auditory response test showed low voltage but normal latency [9]. Another case report in Japan presented a patient with BBE that showed prolonged wave latency in BAER which was similar to our patient [10].

**Table 4:** Diagnostic criteria for Bickerstaff brainstem encephalitis * Lateral symmetry is the rule but mild laterality is also permitted + Features other than the incomplete item(s) must meet (1)

Diagnostic criteria for Bickerstaff brainstem encephalitis [3]
"Definite" BBE is defined when (1), (2), and (4) are satisfied.
"Probable" BBE is defined when (1) and (4) or when (2), (3), and (4) are satisfied.
(1) Acute progressive external ophthalmoplegia,* ataxia, and impaired conscious level by four weeks, followed by spontaneous recovery within 12 weeks after onset.
(2) Positive for serum IgG anti-GQ1b antibodies.
(3) Incomplete agreement on (1) because of one or more of the following reasons+: (a) It is impossible to evaluate ataxia because of severe limb weakness or consciousness disturbance. (b) Unconfirmed recovery of the symptoms. (c) Remarkable laterality of external ophthalmoplegia. (d) Long tract sign (hemisensory disturbance, pyramidal sign, or spasticity) instead of impaired level of consciousness.
(4) Other conditions are excluded in laboratory and image tests: The excluded conditions are Wernicke encephalopathy, cerebrovascular disorder, multiple sclerosis, neuromyelitis optica, neuro-Behcet syndrome, neuro-Sweet disease, pituitary apoplexy, viral brainstem encephalitis, myasthenia gravis, brainstem tumor, vasculitis, botulism, and Hashimoto encephalopathy.

There is a lack of consensus on the treatment of BBE because of its rare occurrence and its natural course of spontaneous recovery. Due to the unavailability of clinical trials, the Cochrane systematic review published in 2007 did not provide evidence-based clinical practice recommendations on managing this disease [11]. Owing to its hypothesized mechanism, immunomodulatory therapies such as plasma exchange, IVIG, steroids, or a combination of these agents are frequently used. However, there were severe cases that failed to respond to the treatments mentioned above. A case study published by Hardy et al. reported a 27-year-old male with severe BBE who was seropositive for anti-Gq1b antibody. He showed poor response to a combination of treatments including IVIG, plasma exchange, and high-dose steroids. He was given rituximab treatment and showed significant neurological improvement a few days after infusion [12]. Our patient also had a rapidly progressive course, had an insufficient response to IVIG and high-dose steroids, and started clinical improvement several days after rituximab was given.

Rituximab is a chimeric IgG1 monoclonal antibody against CD20, a human B-lymphocyte antigen. It is initially approved for the treatment of non-Hodgkin B-cell lymphomas. IgG1 is the most abundant subclass in the body with a long half-life of 21 days. It is produced in response to soluble protein antigens and membrane proteins. Together with IgG3, they are potent triggers of effector mechanisms of adaptive immunity. This is the reason why most cell-depleting monoclonal antibody therapies are IgG1 antibodies [13]. Rituximab's mechanism is complex with several theories explaining its efficacy in various autoimmune diseases. Primarily, it is thought to mediate both antibody- and complement-dependent immune responses resulting in CD20-positive B-cell depletion. In addition, rituximab has been shown to have other immunomodulatory effects by decreasing early T-helper cell activation [14] and by producing rituximab-opsonized B cells which serve as decoy immune complexes that divert monocytes or macrophages from interactions with tissue-associated immune complexes [15]. In a study by Halstead et al., they showed anti-GQ1b antibodies binding and disrupting presynaptic motor nerve terminals at the neuromuscular junction in vitro mouse models with MFS. The mechanism for the injury is through activation of the complement system, leading to the formation of membrane attack complex in nerve membranes [16]. This process may be the pharmacotherapeutic target of several immunomodulatory drugs such as rituximab and eculizumab for the treatment of BBE. Despite its proven benefit in various autoimmune diseases and hematologic malignancies, there were several case reports on the occurrence of GBS after rituximab therapy. Two of the patients had non-Hodgkin lymphoma, and the third had idiopathic thrombocytopenic purpura; all of them received rituximab before the onset of neurologic symptoms and had no preceding infection [17-19]. It has been suggested that this paradoxical response may be secondary to the dysregulation and loss of feedback mechanism during CD20 downregulation with rituximab [19]. However, there are also reports that these diseases themselves were associated with the occurrence of GBS. Whether these are just temporal coincidences, or there is a real association between rituximab and GBS, further studies are needed to investigate this phenomenon.

Despite the alarming clinical presentation and rapid course of the disease, patients with BBE generally have a good prognosis. A study done by Yoshikawa et al. showed that there was no significant difference in the treatment response, disease severity, and prognosis between anti-GQ1b antibody-positive and anti-GQ1b antibody-negative cases. However, it was observed that consciousness disturbance resolved earlier among anti-GQ1b-positive cases (10 days vs 23 days) [1]. Furthermore, in a case series of 62 patients studied by Odaka et al., 66% had complete remission without residual symptoms after six months, while three patients died during illness. Among those with residual neurologic deficits, the most common symptoms were dysesthesia, limb weakness, diplopia, and gait disturbance [2].

## Conclusions

In limited resource settings where serologic testing is not readily available, electrophysiologic testing such as BAER can be used to help diagnose BBE. This is particularly helpful in cases when neuroimaging fails to demonstrate lesions in the brainstem.

A definitive treatment for BBE is yet to be established. There is promising evidence on the use of rituximab as a treatment for severe cases who failed to respond to conventional treatment, warranting further studies to support its use in the treatment of BBE.
